# A Brain-Computer Interface for Improving Auditory Attention in Multi-Talker Environments

**DOI:** 10.1109/access.2025.3623842

**Published:** 2025-10-20

**Authors:** STEPHANIE HARO, CHRISTINE BEAUCHENE, THOMAS F. QUATIERI, CHRISTOPHER J. SMALT

**Affiliations:** 1 Human Health and Performance Systems Group, MIT Lincoln Laboratory, Lexington, MA 02421, USA; 2 Speech and Hearing Bioscience and Technology, Harvard Medical School, Boston, MA 02115, USA; 3 School of Engineering, Brown University, Providence, MA 02912, USA

**Keywords:** Auditory attention, auditory attention decoding, brain-computer interface, electroencephalogram, speech perception

## Abstract

There is significant research in accurately determining the focus of a listener’s attention in a multi-talker environment using auditory attention decoding (AAD) algorithms. These algorithms rely on neural signals to identify the intended speaker, assuming that these signals consistently reflect the listener’s focus. However, some listeners struggle with this competing talkers task, leading to suboptimal tracking of the desired speaker due to potential interference from distractors. The goal of this study was to enhance a listener’s attention to the target speaker in real time and investigate the underlying neural bases of this improvement. This paper describes a closed-loop neurofeedback system that decodes the auditory attention of the listener in real time, utilizing data from a non-invasive, wet electroencephalography (EEG) brain-computer interface (BCI). Fluctuations in the listener’s real-time attention decoding accuracy were used to provide acoustic feedback. As accuracy improved, the ignored talker in the two-talker listening scenario was attenuated; making the desired talker easier to attend to due to the improved attended talker signal-to-noise ratio (SNR). A one-hour session was divided into a 10-minute decoder training phase, with the rest of the session allocated to observing changes in neural decoding. In this study, we found evidence of suppression of (i.e., reduction in) net neural tracking and decoding of the unattended talker when comparing the first and second half of the neurofeedback session (*p* = 0.02, Cohen’s *d* = −1.29, 95% CI [−0.02, −0.01] and *p* = 0.01, Cohen’s *d* = −1.56, 95% CI [−7.25, −3.44], respectively). We did not find a statistically significant increase in the neural tracking or decoding of the attended talker. These results establish a single session performance benchmark for a time-invariant, non-adaptive attended talker linear decoder utilized to extract attention from a listener integrated within a closed-loop neurofeedback system. This research lays the engineering and scientific foundation for prospective multi-session clinical trials of an auditory attention training paradigm.

## I. INTRODUCTION

To perceive spoken human speech, the auditory processing pathway first neurally encodes the acoustic and articulatory characteristics of the stimulus. In environments with noise or multiple talkers, tracking a speech stream becomes more challenging as distractor stimuli are concurrently encoded within the auditory pathway [1], [2]. A listener’s auditory attention serves to help distinguish between neural representations of the attended and distractor streams. Neural activity in response to these acoustic streams have a strong temporal alignment with stimulus features that can be leveraged for decoding purposes [3], [4].

Auditory attention decoding (AAD) is a computational modeling technique used to identify the specific stream to whom a listener directs their attention. This process relies on variations in how each stimuli is represented in the listener’s cortical signals in the form of response strength, temporal characteristics, and topographical features [5], [6], [7]. Listeners have been shown to have heightened encoding of the stimulus of interest relative to the distractor [1], [2], [7]. Listener auditory attention has been been characterized using neural measures that track competing talkers in the scene [7], [8], [9], [10].

An important application of AAD lies in a brain-controlled hearing aid designed to acoustically remix the auditory scene based off of a listener’s desired stream of interest [11], [12], [13]. A brain-controlled hearing aid could use AAD-derived attention tracking metrics as a control signal to facilitate the acoustic enhancement of a given talker’s speech stream [14]. Unlike conventional hearing aids that employ frequency-dependent amplification, a brain-controlled hearing aid offers talker-stream specific remixing within speech-rich environments that often challenge traditional devices [14], [15]. This approach improves the signal-to-noise or distortion ratio of the listener’s desired talker in multi-talker settings, potentially reducing listener effort, improving intelligibility, and elevating the quality of life for individuals across a broad spectrum of age and hearing impairment [16], [17], [18].

For a brain-controlled hearing aid to be feasible in real-world use, it must achieve reliable decoding performance under criteria that differ from those of traditional scientific studies. First, because electrode placement and signal statistics vary from day to day, calibration time must be kept short compared to what is traditionally used in experimental studies [19]. Second, the system must operate in real time with minimal latency in order to track rapid shifts of auditory attention [20]. Third, robustness across listeners with diverse audiometric profiles is essential, rather than restricting validation to young, normal-hearing cohorts commonly studied in experimental studies. Many proof-of-concept AAD studies have reported statistical effects that fail to translate to end-user benefit, owing to small effect sizes, long training requirements, or limited generalizability. In this study, we explicitly address these feasibility criteria by limiting decoder calibration to 10 trials (10 minutes), implementing a real-time feedback loop with 1 s latency, and recruiting a cohort spanning a range of ages and audiometric profiles, thereby situating our work closer to translational applicability.

Unfortunately, there exists a broad range in the performance of AAD algorithms across the population, which limits the benefit that could be gained from an AAD-driven brain-controlled hearing aid. It is not known whether hardware interface differences may contribute to poor AAD performance, which could include electrode impedance, or physical geometrical factors specific to an individual. Cochlear damage, i.e., poor pure-tone audiometry, would be an obvious source of poor AAD performance, but at least one study has found greater performance in hearing impaired individuals [21]. Another possibility that could explain poor AAD is a listener’s difficulty in managing their cognitive resources to regulate their attention [2]. This deficiency in attentional capability is likely to result in poor neural tracking measures which cannot be ameliorated by advances in AAD hardware personalization or algorithmic engineering. Consequently, it may be imperative to enhance the robustness and quality of a listener’s attention neural tracking measures in order to make them a candidate for a brain-controlled hearing aid. Significant work remains to advance decoding models via machine learning techniques; however, improving decoding through human adaptation is a relatively unexplored domain. This study introduces an auditory attention training paradigm aimed at helping listeners increase auditory attention control through a closed-loop neurofeedback process.

Closed-loop systems are gaining prominence in various neuroscience domains as they seek to take advantage of training paradigms for the investigation of learning processes. Auditory training paradigms have shown efficacy in improving speech processing in multi-talker environments by improving source segregation, perception of weak target signals in the midst of competing stimuli, or language processing [22], [23], [24]. These auditory training paradigms demonstrated preliminary success in improving speech-in-noise perception in multi-talker scenes, but the results differ in the neural basis, i.e., the underlying neurological mechanisms, attributed to the task improvement.

**FIGURE 1.**
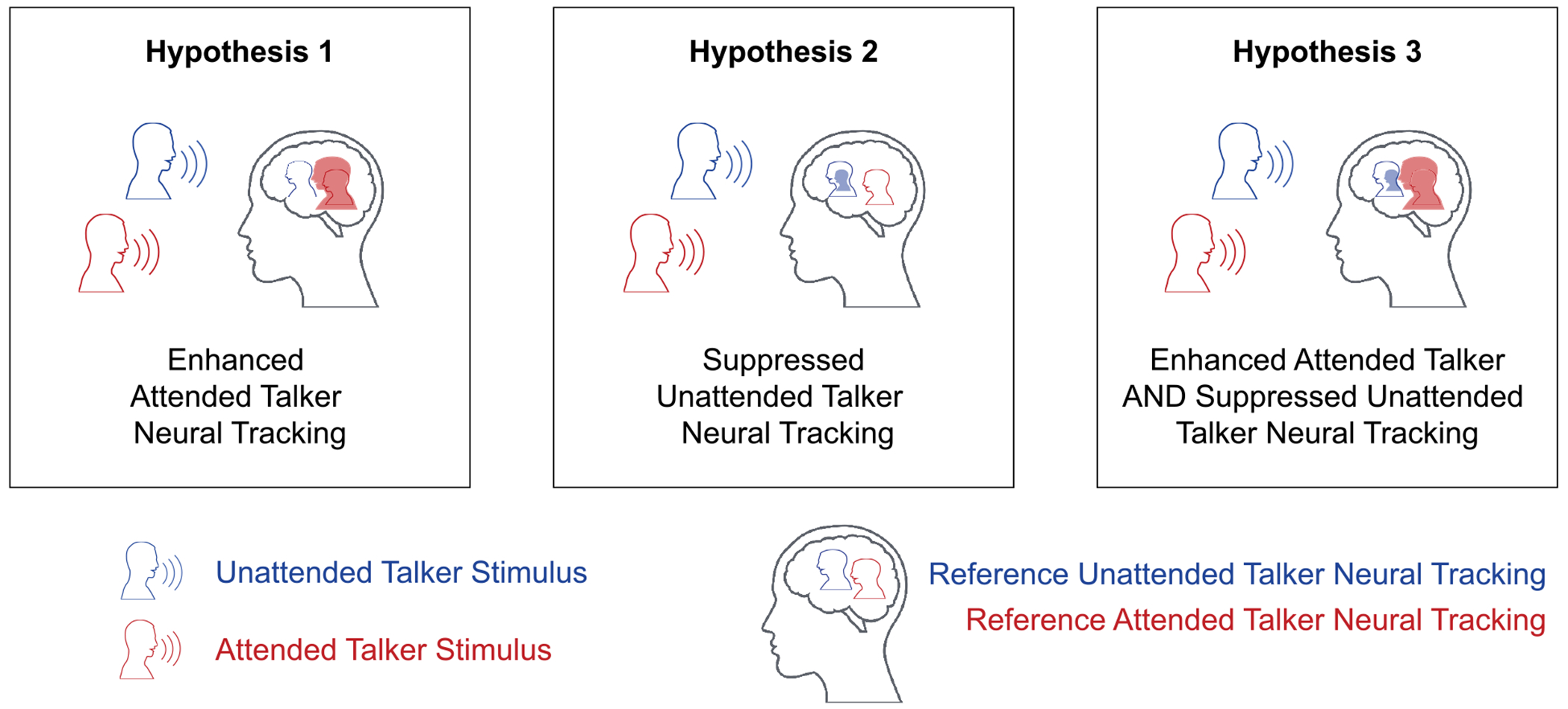
The AAD-driven neurofeedback paradigm’s goal is improved auditory attention decoding of the desired talker stream; it may be attained through three neural mechanisms. *Hypothesis 1:* Enhanced neural tracking of the desired attended talker, *Hypothesis 2:* Suppressed neural tracking of the unattended talker, or *Hypothesis 3:* A combination of enhanced neural tracking of the attended talker and suppressed neural tracking of the unattended talker.

This study developed a real-time system that monitors a listener’s brain activity to identify the attended talker of interest and dynamically uses this neural indicator to amplify that talker’s acoustic stream. This approach aims to help listeners focus on a target talker in noisy multi-talker environments, while also determining the neural mechanisms underlying improved auditory attention. We define improved attention as changes in neural activity reflecting the attention-driven encoding of auditory streams in the scene. Using a developed real-time AAD-driven neurofeedback paradigm, this work tested three potential neural mechanisms for improving auditory attention. These three hypotheses were evaluated by examining changes in attention decoding performance across the protocol (Figure 1). *Hypothesis 1:* Enhanced listener attention arises from increased neural tracking of the attended talker, evidenced by improvements in attended talker decoding accuracy. *Hypothesis 2:* Improved attention may result from suppressed neural tracking of the unattended talker, reflected by reductions in unattended talker decoding accuracy. *Hypothesis 3:* Attention enhancement may involve a combination of both increased attended and suppressed unattended neural tracking. This investigation of real-time feedback-driven improvements in auditory attention lays the foundation for future multi-session paradigms designed to enhance the performance and practical utility of brain-controlled hearing aids.

## II. METHODS

### A. PARTICIPANT PREPARATION AND SIGNAL RECORDING

A total of 24 participants (10 females, 14 males) were initially enrolled in the study protocol. However, two participants were excluded from the cohort due to inadequate pupil diameter measurements obtained during the neurofeedback procedure. Consequently, the final participant cohort was composed of 22 native English speakers (9 females, 13 males). All participants provided informed consent for participation in the experimental protocol, which received approval from the MIT Committee on the Use of Humans as Experimental Participants and The U.S. Army Medical Research and Development Command, Human Research Protection Office.

Participants were situated in a sound-treated booth for the neurofeedback paradigm protocol. They were seated equidistantly between two loudspeakers positioned at a 45-degree angle, located approximately six feet away. During the experiment, the left and right loudspeakers presented male talkers reading the audio books “Twenty Thousand Leagues Under the Sea” and “Journey to the Center of the Earth,” respectively [10]. Participants were instructed on the protocol using a standardized script that included guiding them on how to minimize artifacts in their EEG and pupil diameter data throughout the experimental segments designated for analysis. A computer monitor placed directly in front of them was utilized to present text associated with the tasks. Participants were fitted with a 24-channel wet Brain Products EasyCap EEG cap, which wirelessly transmitted data via an mBrainTrain Smarting Bluetooth transmitter. EEG data was acquired at a sampling rate of 500 Hz. Both the stimulus waveforms and EEG recordings were pre-processed in a manner required for real-time AAD (see supplemental materials).

### B. AUDITORY ATTENTION NEUROFEEDBACK PARADIGM OVERVIEW

**FIGURE 2.**
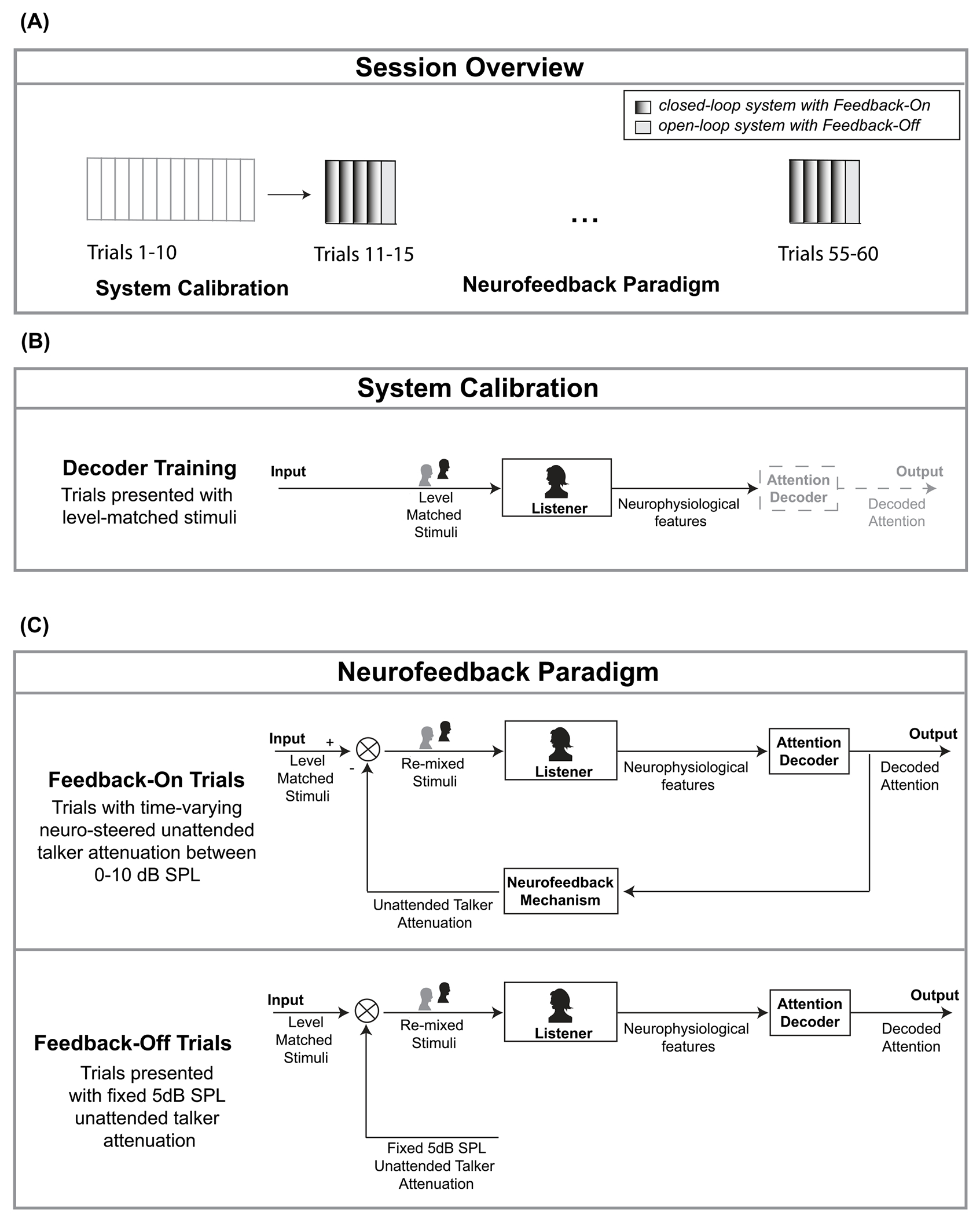
Neurofeedback paradigm overview and schematics (A) The experimental protocol was composed of two phases – a decoder training phase and a neurofeedback paradigm phase. (B) During the first phase, data was collected that was used to quickly train an individualized attended talker decoder during the session. (C) The neurofeedback paradigm phase consisted primarily of trials with closed-loop feedback interspersed with trials that had open-loop fixed unattended talker gain.

The experimental protocol consisted of two distinct phases: an attention decoder training phase and a neurofeedback paradigm phase (Figure 2A). During the initial phase, data from 10 one-minute trials were collected and used to train an attended talker attention decoder tailored for each participant (Figure 2B). During the training phase, all participants were subjected to a balanced presentation of trials in which participants were directed to attend alternately to the left and right talkers. The participants were told that the initial 10 trials would be used to calibrate their individualized attention decoder. We communicated that a check-in would occur after the completion of the initial 10 trials to provide further instructions. In these subsequent instructions, we indicated that the participant would be asked to attend to a single randomly selected talker for the remainder of the session. We balanced the number of participants that attended to the left and right talker for the remainder of the paradigm. The decoder was employed in the subsequent 50 trials to drive the neurofeedback that the participant received for the rest of the session. This neurofeedback phase of the protocol predominantly involves feedback-on trials, with dynamically changing unattended talker gain, that were interspersed with trials that had a fixed unattended talker gain (Figure 2C). Participants were told that we would provide periodic progress updates, although the specific trial number would not be disclosed upon request. Unbeknownst to the participants, the neurofeedback was inhibited every fifth trial, during which the unattended talker’s audio was consistently attenuated to a fixed level. These trials with fixed gain were included to disentangle temporal effects as the session elapsed from listener changes caused by the neurofeedback mechanism. Between trials, participants were required to respond to comprehension questions regarding the attended talker’s audio book. These questions functioned as a behavioral measure of the participant’s comprehension regarding the attended talker [10].

### C. CLOSED-LOOP NEUROFEEDBACK PLATFORM

To implement the neurofeedback paradigm it was necessary to develop a closed-loop AAD system capable of providing real-time feedback to the user. Such a system is not commonly employed in conventional auditory attention decoding experiments, as decoder training and analyses are typically conducted offline utilizing all the trials of data gathered during an experiment. In the conventional open-loop design, these experimental and analysis stages occur sequentially: the investigator initiates the recording; the participant proceeds through a predetermined stimulus presentation and data is collected during the experiment, data is stored in a file system when the investigator terminates the recording and exports the data files and lastly, the investigator loads the exported files into offline analysis scripts [25], [26]. Our closed-loop system necessitated a revision of these steps to enable the recorded data to interface directly with the experimental protocol, effectively placing the user within a neurofeedback loop. The majority of decoding algorithms have historically been executed offline, and only recently have real-time algorithmic considerations been explored with the eventual aim of developing a functional real-time system [24], [27], [28], [29]. To facilitate our auditory training paradigm involving real-time auditory attention decoding, the system was designed to operate in a closed-loop manner, and required data collection, decoding, and feedback to occur in real-time without the need for external investigator intervention, which included pre-processing and time synchronization. The overall delay of the system is approximately 6.1 s due to the approximately 1.1 s delay associated with the causal pre-processing steps performed on the EEG and audio data, along with a 5 s delay associated with detecting a possible switch in attention using the 10 s long decoder correlation window (see supplemental materials). We assert that this closed-loop system operates with precision and accuracy that fulfills the protocol’s requirements. This work is among the few to evaluate these algorithms in a truly real-time, causal manner [19].

### D. AUDITORY ATTENTION DECODING

To decode attention, we opted for a temporal-response-function (TRF) based method that involved training a decoder using samples of continuous, non-repeating trials of speech. There exists many non-linear AAD modeling approaches [28], [30], [31], [32], [33], which aim to optimize decoding reliability, albeit often at the expense of necessitating substantial training datasets and prolonged training time that are impractical for immediate deployment in a brain-controlled hearing-aid context. Another popular method used by the field is a correlation-based linear attention decoding approach, which uses a linearly decoded attended talker time series, coupled with a correlation step that is used to ascertain the listener’s attended talker from two candidate talker envelopes in the acoustic scene. This linear approach is favored for its rapid training in situations where data quantity is limited. This linear decoding method can be configured to decode features of either the attended or the unattended talker stimuli, making it useful as a neural tracking measure tool of any talker in the scene. For the neurofeedback paradigm, the attended talker was selected due to its higher accuracy compared to the unattended decoder. The neurofeedback paradigm employed an individualized attention decoder for each participant, which was trained during the calibration phase encompassing the initial 10 trials of the session. The decoder was trained under data-limited conditions and utilized only 10 minutes of data, which diverges from typical AAD practices that employ substantially larger datasets [10], [31], [34]. The attended talker decoder underwent training in an online manner, specifically while the participant was responding to comprehension questions between trials 10 and 11 within the protocol.

The decoder was configured with 24 channels and spanned 500 ms in duration. The listener’s neural data used for training, $N_{Tr}$, was constructed with a balanced amount of data reflecting instances when the listener attended to a specified talker and then attended to the secondary talker present in the auditory scene (Eq. 1). The attended talker decoder, $W_{Att}$ was solved using L2 (ridge regression) regularized least squares using a regularization parameter of 1e2 that was selected based off of a pilot cohort (Eq. 1). Should the weights of the attended decoder, $W_{Att}$, be applied to the test window of neural data, $N_{\mathrm{Test}}$, the resultant prediction is the attended talker envelope, ${\hat{env}}_{Att}$ (Eq. 2).

(1) $$W_{Att}={\left({N_{Tr}}^{T}N_{Tr}+\lambda I\right)}^{-1}\left({N_{Tr}}^{T}{Env}_{Att,Tr}\right)$$

The attended and unattended talker envelopes are predicted from a segment of neural data, $N_{\mathrm{Test}}$, using the corresponding talker decoder:

(2) $${\hat{env}}_{\mathrm{Att}}=N_{\mathrm{Test}}W_{\mathrm{Att}}$$

The decision regarding the attended talker was determined using the attended decoder, as outlined through a sequence of steps specified in Eq. 3–6. First, the predicted attended talker envelope, denoted as ${\hat{env}}_{Att}$, was subjected to a Pearson correlation with the true candidate talker envelopes, which have been ideally separated into distinct streams ${env}_{Att}$ and $env_{Una}$, respectively (Eq. 3–4). Metric ${\mathrm{corr}}_{Att,Att}$ measures the decoded attended envelope’s neural similarity with the the attended talker envelope. Conversely, metric ${\mathrm{corr}}_{Att,Una}$ measures the predicted attended envelope’s similarity with the unattended talker envelope. The stronger Pearson correlation among the two indicates which talker the predicted attended envelope most closely resembles. Metric ${\mathrm{corrDiff}}_{\mathrm{Att}}$ assesses the disparity between ${\mathrm{corr}}_{Att,Att}$ and ${\mathrm{corr}}_{Att,Una}$, highlighting the unique neural tracking to the attended talker envelope that is not shared with the unattended talker envelope. The fraction of samples where ${\mathrm{corrDiff}}_{\mathrm{Att}}$ is greater than zero signifies the decoder’s accurate determination of the attended talker (Eq. 6). The unattended decoder was trained offline at the completion of the session, utilizing the same 10 minutes of data that were employed to train the attended talker decoder but with inverted labels (supplemental materials). A similar process was performed to train the unattended decoder and derive the unattended talker decoder correlation metrics (supplemental materials). Both decoder’s output measures can be used to quantify neural tracking of the attended and unattended talkers in the scene (Table 1). The direction in which these measures change over the course of the session have neural tracking implications that contribute towards a listener’s attention.

(3) $${\mathrm{corr}}_{Att,Att}=\mathrm{corr}\left({\hat{env}}_{Att},{env}_{Att}\right)$$

(4) $${\mathrm{corr}}_{Att,Una}=\mathrm{corr}\left({\hat{env}}_{Att},{env}_{Una}\right)$$

(5) $${\mathrm{corrDiff}}_{Att}={\mathrm{corr}}_{Att,Att}-{\mathrm{corr}}_{Att,Una}$$

(6) $${acc}_{Att}=\mathrm{mean}\left({\mathrm{corrDiff}}_{Att}>0\right)$$

### E. NEUROFEEDBACK MECHANISM

**FIGURE 3.**
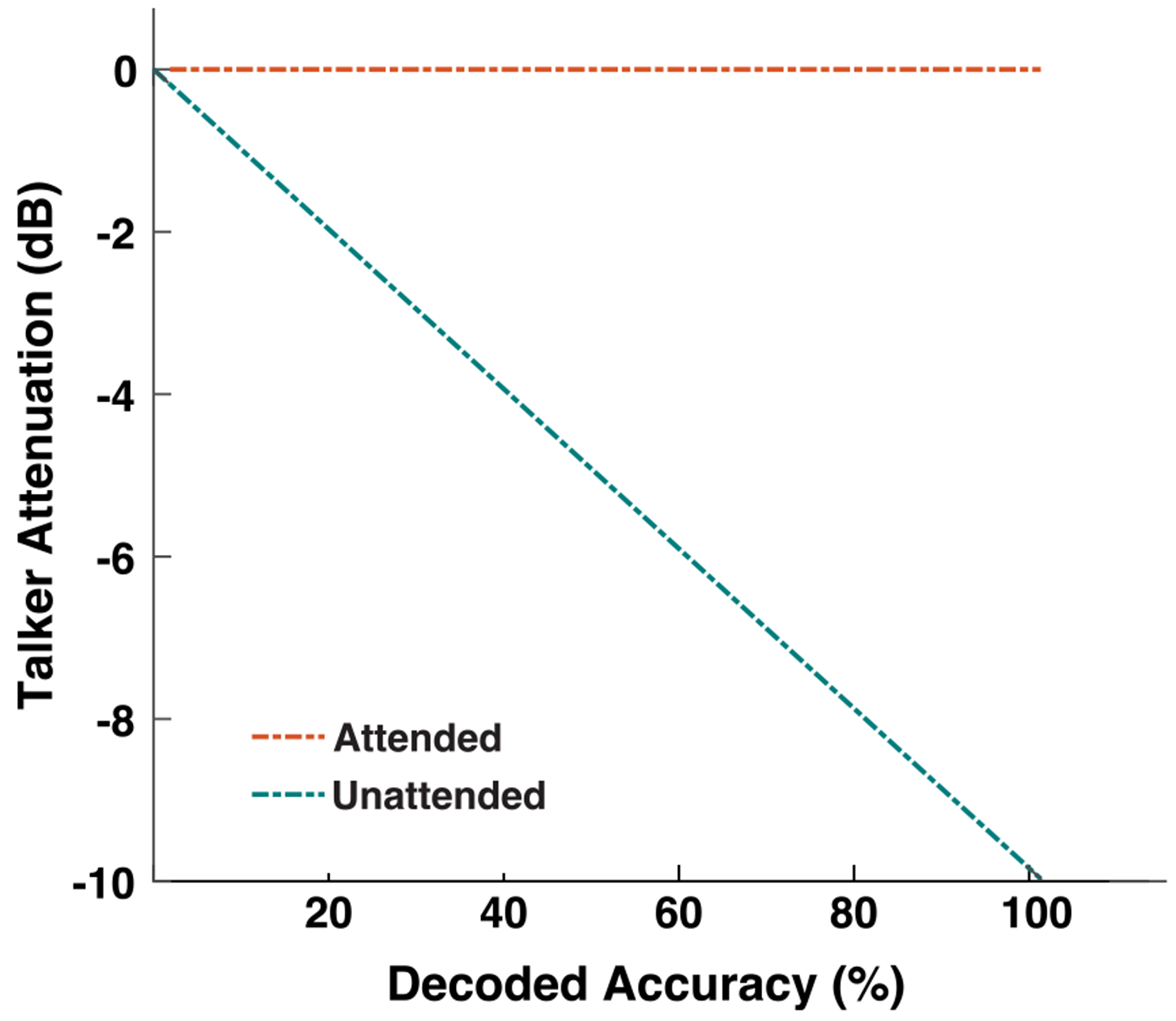
The neurofeedback mechanism applied an inverse relationship between the attended talker decoding accuracy and the unattended talker presentation level. The system updated every 500 ms.

In the neurofeedback paradigm, the feedback mechanism employed a smoothed version of the listener’s real-time, time-variant decoded attention signal to augment the level of unattended talker attenuation. Figure 3 depicts this inverse relationship between the attended talker decoding accuracy and unattended talker attenuation level. The attenuation of the unattended talker ranged from 0 to −10 dB SPL, responding to a real-time decoded attention accuracy spanning from [0,100]. The presentation level of the attended stimulus remained constant; thus, as the unattended talker stimulus level diminished, the signal-to-noise ratio (SNR) between the attended and unattended talker increased. Participants were presented with greater distractor attenuation in response to improved attended talker decoding accuracy. This in turn created an auditory scene where the desired talker becomes easier to sustain attention towards [35]. For each trial, unattended talker attenuation was quantified as the integral of attenuation normalized to the trial’s maximum possible attenuation (Eq. 8).

(7) $$\alpha_{\max}=\int_{1}^{N}-10\;dB\;SPL(t)dt,\;t\in \text{samples in trial}$$

(8) $$\alpha_{deg}=\frac{1}{\alpha_{\max}}\int_{1}^{N}\alpha (t)dt,\;t\in \text{samples in trial}$$

**FIGURE 4.**
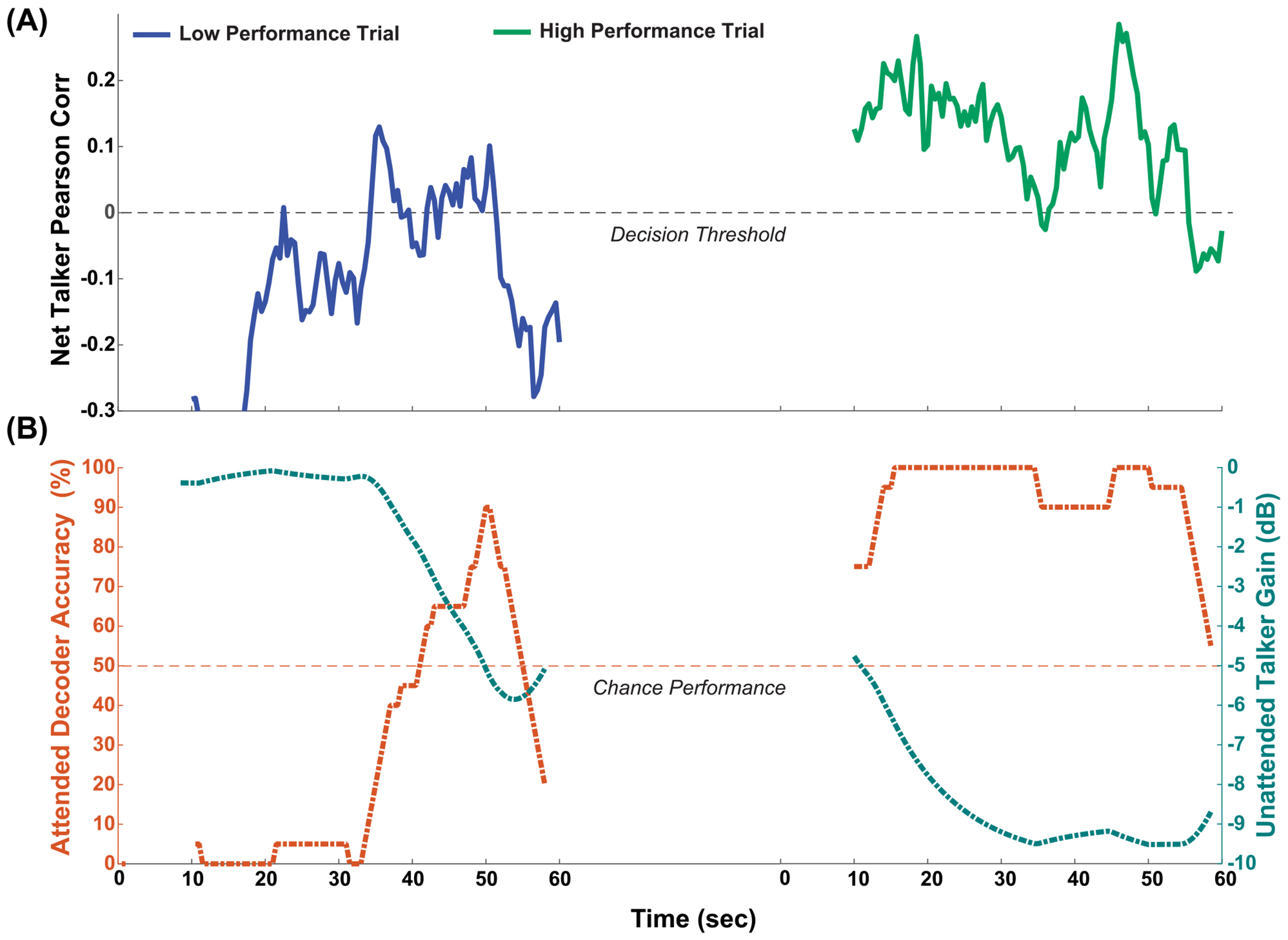
A listener’s auditory attention dynamics visualized across a low and high performing performing trial. The top panel plots the attended talker decoder measure, ${\mathrm{corrDiff}}_{A}$, which indicates the net attended talker decoded neural similarity to the two talker stimuli. The bottom panel plots the fraction of ${\mathrm{corrDiff}}_{A}>0$ in the form of attended decoder accuracy along with the unattended talker gain which is driven by the value of the attended decoder accuracy. Listener driven changes in unattended talker gain is the neurofeedback mechanism used in this paradigm.

Auditory attention exhibits significant dynamism within the duration of a single trial which can be visualized through decoder-derived neural tracking measures of the talker stimuli. In Figure 4A the net talker Pearson correlation, ${\mathrm{corrDiff}}_{A}$, is depicted which is derived from the application of the attended decoder to one-minute EEG data segments. The two ${\mathrm{corrDiff}}_{A}$ time series are associated with trials that differ in performance. In the low performance trial on the left, ${\mathrm{corrDiff}}_{A}$ briefly rises above the decision threshold of zero. In the high performance trial on the right, ${\mathrm{corrDiff}}_{A}$ is sustained above the decision threshold for the majority of the time with fluctuations in ${\mathrm{corrDiff}}_{A}$ strength. Figure 4B, illustrates the sliding accuracy of the attended decoder and the teal trace represents the listener-driven unattended talker stimulus gain for the ${\mathrm{corrDiff}}_{A}$ from Figure 4A.

The neurofeedback paradigm alternated between trials during which participants were actively engaged with the neurofeedback and trials where participants were presented with fixed unattended talker gain (Figure 2C). In the session, 80% of the 50 remaining trials involved neurofeedback. Trials are unlabeled, with every fifth trial being a fixed unattended talker gain trial. The fixed unattended talker gain trials employed an open-loop architecture, where it is predetermined that the unattended talker is presented with −5dB SPL of attenuation. We set the unattended talker gain to −5dB, the midpoint of the neurofeedback range, so that participants would be less likely to notice when the gain was not being actively driven during these trials. These fixed unattended talker gain trials were incorporated to facilitate the interpretation of potential effects observed throughout the session in the feedback-on trials. Should there have been a change in neural tracking measures present in the feedback-on trials that is absent in the fixed unattended talker gain trials, such changes could be ascribed to the neurofeedback with which the listener was engaged, rather than the result of mere acclimation to the task over time. Conversely, if changes in neural tracking measures were evident in both feedback-on and fixed unattended talker gain trials, it may be posited that the listener applied learned improved attention techniques to trials that did not contain feedback. An intervention could be deemed successful if there are persistent effects of learning that generalize to situations lacking neurofeedback [23], [36].

### F. STATISTICAL TESTING

In order to assess the differences in measures between the trials with and without feedback, two-factor ANOVA tests were conducted, with the trial type (‘feedback’ vs ‘no feedback’) treated as a fixed factor and the participant treated as a random effect. Similarly, differences between the first and second halves of the session were examined using two-factor ANOVA tests, where the session half (‘first-half’ vs ‘second-half’) was the fixed factor with participant as a random effect. For all t-tests, we report effect sizes using Cohen’s *d* along with 95% confidence interval derived from the t-distribution. In addition, we computed Pearson correlations between the validation and testing accuracy, reporting the correlation coefficient, the associated p-value, and 95% confidence interval.

## III. RESULTS

### A. AUDITORY ATTENTION DECODER PERFORMANCE

**FIGURE 5.**
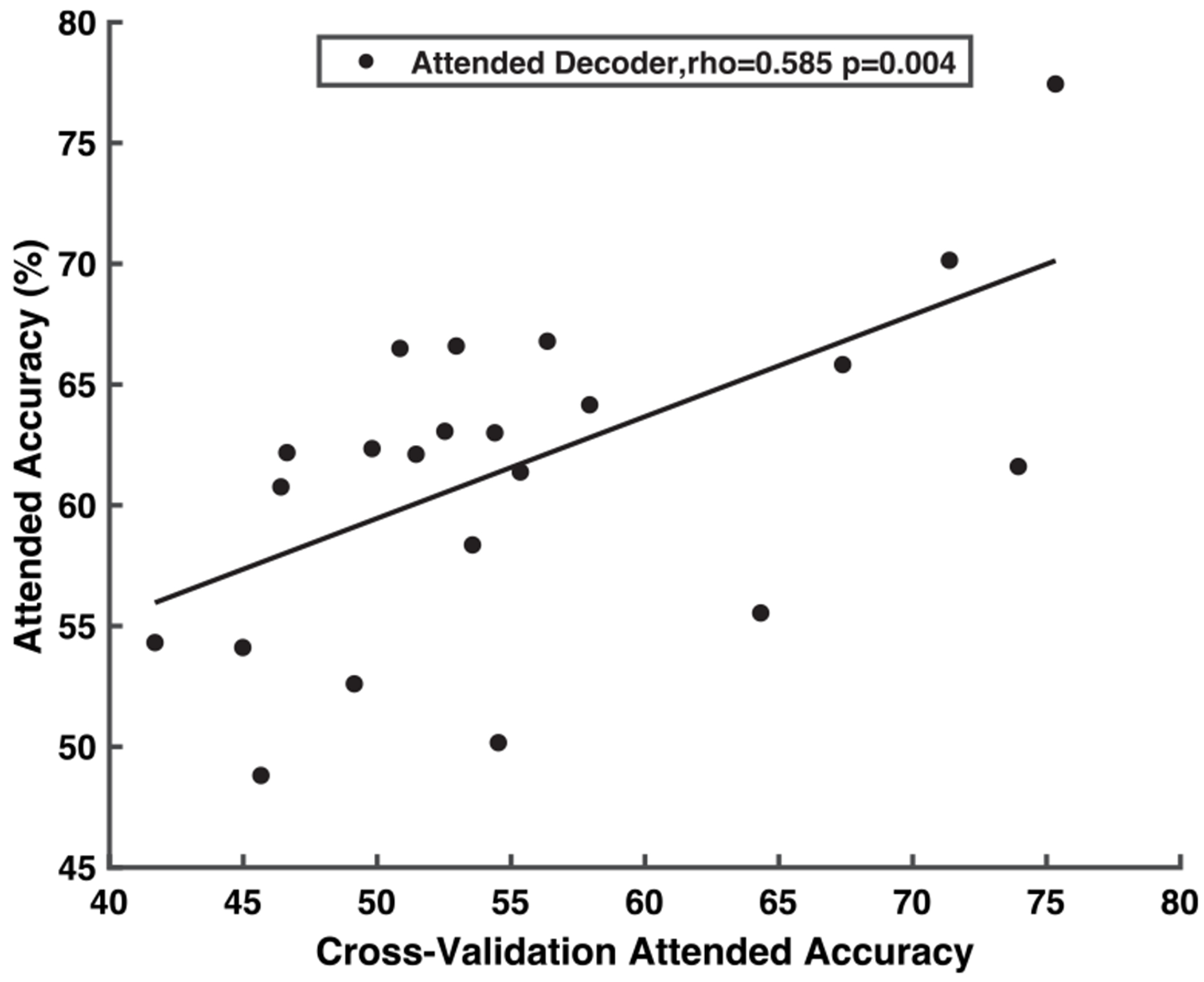
Attended talker decoder accuracy correlated with leave-one-trial out cross-validation accuracy. A participant’s decoded attention accuracy during the test trials is correlated with the accuracy achieved during the decoder calibration phase.

To assess the accuracy of attended decoder training, we performed a cross-validation analysis of leave-one-trial-out using only the first 10 (training) trials. The cross-validation accuracy is represented on the horizontal axis of Figure 5. The mean accuracy of the cohort of the attended decoder was 60.8% (SD = 7.1%). Although this mean accuracy may be regarded as low, it should be interpreted in the context of the limited amount of training data available before deploying the decoder in real-time, the linear decoding method used, and the relatively short correlation-based decision window used to improve decoder reactivity. The accuracy of the decoder trained on the complete 10-minute dataset was evaluated over the remaining 50 trials, as depicted on the vertical axis of Figure 5. As anticipated, a range of decoding accuracies were observed among participants. Moreover, decoded attended accuracy during the test trials was correlated with the accuracy achieved during the decoder calibration phase (ρ=0.59, p = 0.004, 95% CI [0.28,0.81]), indicating that participants with higher calibration accuracy tended to have higher test accuracy.

### B. EXCLUDING OTHER SOURCES OF MODULATION

**FIGURE 6.**
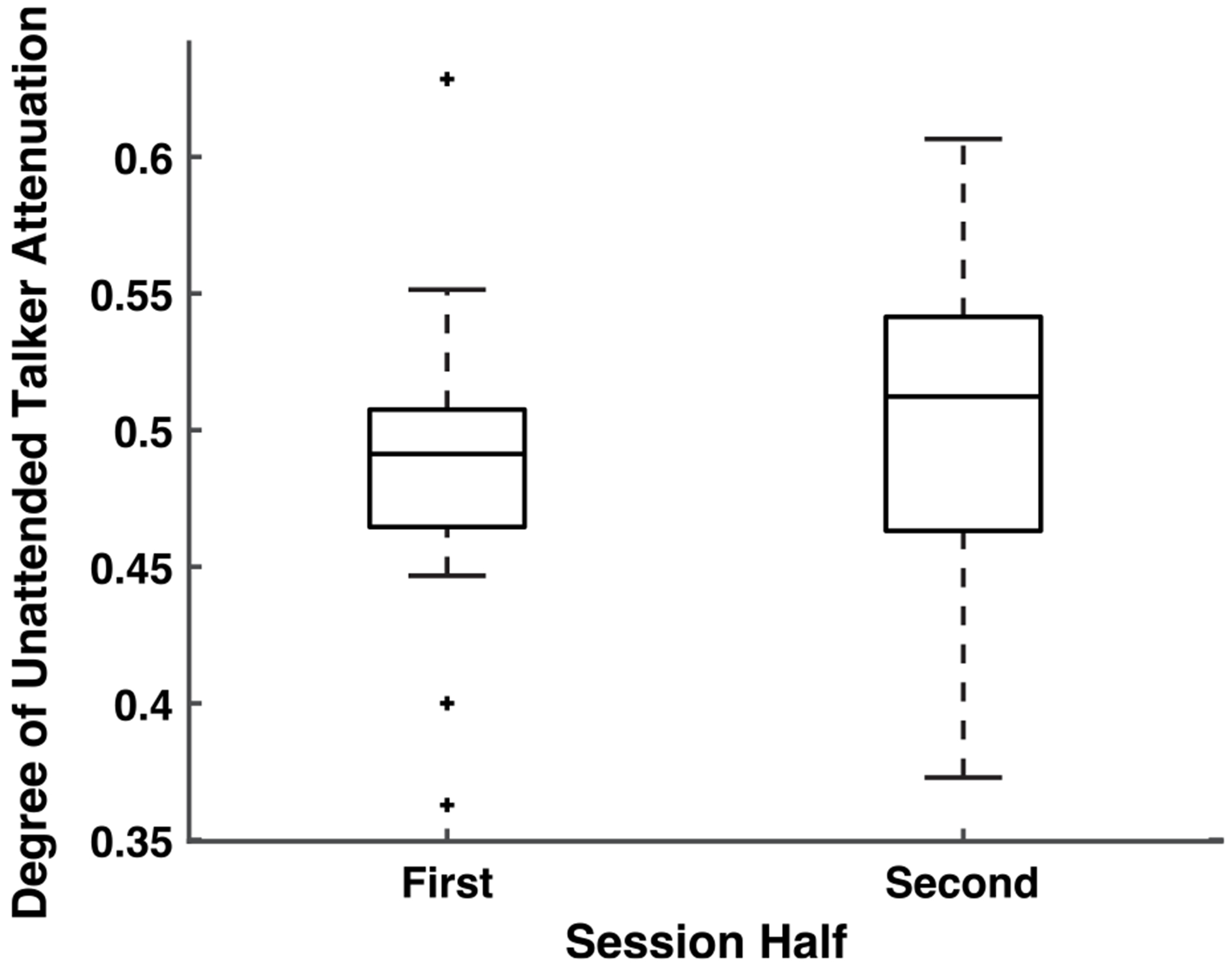
The degree of unattended talker attenuation is not significantly different across the session. Therefore, any differences seen across the session are not due to significantly different talker presentation level differences but instead may represent changing listener neural representations of the talker.

The mean comprehension accuracy across participants was 63.4% (SD = 13.7%) on the four-choice comprehension questions asked at the end of each trial (chance of 25%). It is important to note that these questions are particularly detailed but were used since the stimulus set was available and used for consistency with past studies [10], [20]. Furthermore, we detected no difference in comprehension score accuracy between session halves, suggesting that participants maintained consistent engagement throughout the session. We also did not observe differences in the unattended talker presentation level over the course of the session (Figure 6). This indicates that any significant differences in decoding measures seen were not due to alterations in stimulus presentation characteristics over the session. Since we rule out listener engagement and stimulus presentation level as external sources of change, any changes in attention can be interpreted as being driven by the user being embedded in the feedback system.

### C. NEUROFEEDBACK IMPACT ON AUDITORY ATTENTION

**FIGURE 7.**
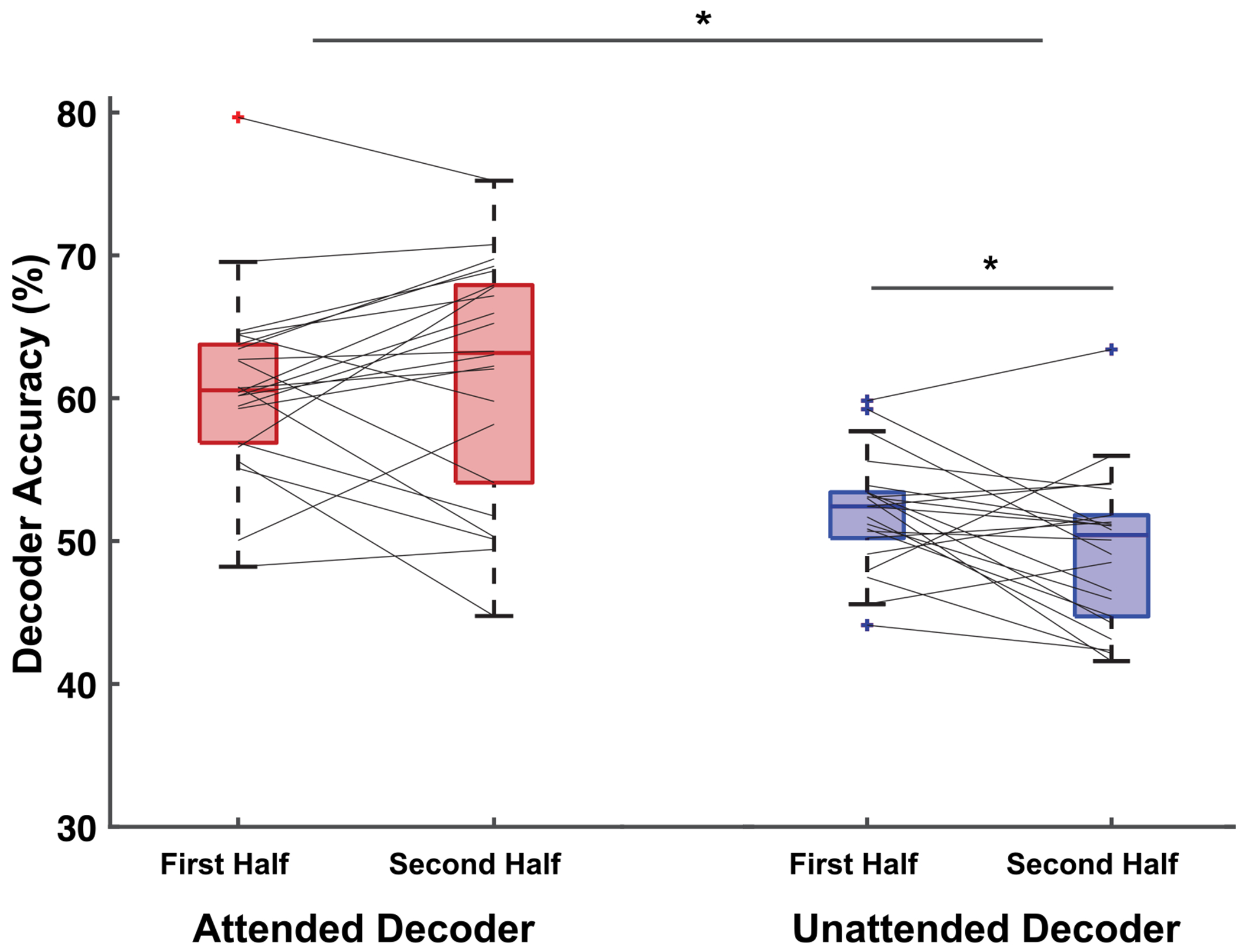
Attended and unattended talker decoder accuracy serve as neural tracking measures of the attended and unattended talker respectively. Differences in decoder accuracy for trials that had feedback present across the session can be used to determine the neural basis of improved attention due to closed-loop neurofeedback. **Hypothesis 1:** Attended talker decoding accuracy, *acc_Att_*, did not change across the session in trials that had feedback present, indicating that attended talker neural tracking was not enhanced in response to the neurofeedback. **Hypothesis 2:** Unattended talker decoding accuracy, *acc_Una_*, decreased across the session in trials that had feedback present, indicating that unattended talker neural tracking was suppressed in response to the neurofeedback. **Hypothesis 3:** Since Hypothesis 1 was rejected, we too can reject Hypothesis 3.

By comparing AAD-derived neural tracking measures across the session, each of the hypothesized neural bases of improved attention were assessed. During trials where feedback was present, there were no substantial differences in the attended talker decoder correlation metric, ${\mathrm{corrDiff}}_{\mathrm{Att}}$, or in decoding accuracy, ${\mathrm{acc}}_{\mathrm{Att}}$, over the course of the session (Figure 7, left). This finding allowed us to refute our first hypothesis, which posited improved attention via improved neural tracking of the attended talker. Conversely, there was evidence supporting Hypothesis 2 through the observation of reduced tracking of the unattended talker (Figure 7, right). In trials with active feedback, both the unattended decoder correlation metric, ${\mathrm{corrDiff}}_{Una}$, and its decoding accuracy, $acc_{Una}$, diminished across the session halves (*p* = 0.02, Cohen’s *d* = −1.29, 95% CI [−0.02, −0.01] and *p* = 0.01, Cohen’s *d* = −1.56, 95% CI [−7.25, −3.44], respectively). Together, these results indicate a large suppression effect of the unattended talker on neural tracking, with the confidence intervals reflecting the negative bounds in both net unattended talker correlation and unattended decoding accuracy, consistent with the expected direction of suppression between the two halves. This reduction is indicative of a suppression effect of the unattended talker in the listener’s neural responses. This effect on unattended talker neural tracking measures is absent in trials with no feedback, further corroborating the presence of suppressed unattended talker neural tracking facilitated exclusively by the neurofeedback mechanism. Figure 6 illustrates decoder accuracy for the feedback versus no-feedback conditions.

## IV. DISCUSSION

### A. AUDITORY ATTENTION DECODER IMPLEMENTATION

In designing the decoder training phase of the protocol, one of our goals was to minimize training duration due to the limited amount of available stimuli and the demanding attentional nature of the task for participants. Ten minutes of training data were deemed sufficient based on a pilot cohort of three participants, yielding approximately 60% leave-one-trial-out cross-validation accuracy, consistent with prior EEG studies [20], [27]. For the 22 participants in this study, mean cross-validation accuracy was slightly lower, likely reflecting the novelty of the task and the range of individual attention capacities captured in the EEG signal. Calibration accuracy was moderately predictive of test performance (*rho* = 0.59, p = 0.004, 95% CI [0.28, 0.81]), suggesting that decoder quality and the participant’s attentional ability influence downstream accuracy later in the protocol. The confidence interval highlights uncertainty in the magnitude of this effect, emphasizing the need for a larger participant cohort to better characterize reliability. This larger cohort should also capture the range of hearing and attentional abilities that this work aims to benefit. It is plausible that certain subjects might have attained better improved decoder accuracy over the course of the session had the decoder training duration been prolonged to achieve a better trained decoder. We suggest that future research employ a larger stimulus set, allowing the decoder training period to be lengthened until a minimum of 60% accuracy in leave-one-trial-out cross-validation is achieved. Future studies might also adopt a sliding training window approach that integrates newly acquired EEG data and continually retrains the decoder to accommodate recent cortical statistics and variations in talker encoding over time [19].

### B. EXCLUDING OTHER SOURCES OF MODULATION

The improved attention seen across the participant cohort suggests a potential reallocation of the listeners’ cognitive resources. One may argue that decoded attention measures may have been influenced by decoder quality, listener engagement, or stimulus characteristics. These alternative sources of modulation were eliminated as contributing factors that could impact attention. While there are advantages to decoders that are continually retrained to adapt alongside shifts in neural drift, we opted to utilize a fixed decoder [19]. Since we used a fixed decoder, trained once that was not retrained as the session progressed, this ensured that any variations in the decoder-derived attention measures were not attributable to updated decoder characteristics. Additionally, we determined there were no neurofeedback-driven stimulus presentation changes across the session that could explain the reduction in unattended talker neural tracking. Lastly, listener engagement, as evaluated by comprehension score accuracy, could have also modulated over time, potentially affecting attention but we also did not see differences across the session. Therefore, the neural tracking measures that are significant over the session can be interpreted as being driven by true improved attention when taken in combination with the lack of the effect in the trials without feedback.

### C. NEUROFEEDBACK IMPACT ON AUDITORY ATTENTION

The design of out feedback paradigm may have influenced the neural basis of improved attention when users engaged with the paradigm. A limited number of feedback training paradigm studies exist that employ various sensory-feedback combinations, attributing auditory perception modulation to distinct neural bases. Our study design can be characterized as an audio-neural feedback scheme, as it utilizes neural signals to induce a change in the distractor stimulus, which is acoustically perceived by the participant. We observed evidence of suppressed net neural tracking of unattended talkers and an absence of a significant difference in the degree of unattended talker attenuation. The suppression effect was large, and the confidence intervals for unattended accuracy were consistently negative, supporting the reliability of the effect, though replication in a larger cohort would further strengthen this conclusion. This finding corroborates a previous multi-session audio-motor feedback training paradigm, wherein the presentation level of the stimulus of interest was maintained constant [23]. They proposed their paradigm facilitated the participants’ ability to suppress the distractor since the feedback scheme adjusted the distractor stimulus level. In contrast, another multi-session visual-neural paradigm maintained constant levels of competing talker stimuli presentation and found evidence of enhanced attended talker neural tracking over a multi-session study [36]. Consequently, the neural bases we associate with improved attention must be considered within the context of the neurofeedback mechanism design choices we implemented. Lastly we will note that utilizing an unattended talker decoder as the feedback mechanism might have provided greater learning potential throughout the session; but this option was not selected due to the generally inferior accuracy of the unattended decoder [7].

Additionally, for the neurofeedback paradigm, either an attended or unattended decoder could have been used to drive stimulus level changes. However, it is noted that a decoder designed to determine the attended talker will produce a more prominent attended talker neural tracking response than the unattended talker decoder’s produced unattended talker neural tracking measure. This can be attributed to the listener’s heightened encoding of the stimulus of interest relative to the distractor [1], [2], [7]. We considered reducing SNR in response to successful attended talker decoding, but did not choose this design choice, as our aim was not to train ‘super attenders’ capable of maintaining focus on a desired stream at low SNR levels. While there is merit in pursuing this latter objective, it was not the focus of this project. The range of attenuation was used because it still permitted the listener to latch onto the other talker if desired, and this SNR falls in the range that has been used in previous investigation of the impact of stimulus SNR levels on listener effort [37], [38].

### D. TOWARDS CLINICAL AND BCI TRANSLATION FOR IMPROVED AUDITORY ATTENTION

This study serves as a proof of concept for a single session that could be part of a multi-session auditory attention training paradigm. Typically, preliminary research precedes a clinical trial to demonstrate that an intervention, such as neurofeedback, exerts an influence on an outcome (such as a change in attention tracking measures or SIN accuracy) within an experimental group prior to including a control group. To enable translation to a real-time brain-controlled hearing aid, an AAD system must reliably decode attention with sufficient accuracy to allow perceptible improvements in the signal-to-noise ratio of the desired talker. Prior studies suggest that decoder accuracies below approximately 60% often fail to produce meaningful perceptual benefits [13], [14]. In this study, the mean attended decoder accuracy of 60.8% (SD = 7.1%) not only meets but slightly exceeds this benchmark, providing evidence that neural attention signals from a wide range of listeners are sufficient to drive real-time feedback. Achieving this level of performance with only 10 minutes of training and a non-invasive 24-channel EEG cap highlights the practical feasibility of our approach, especially when compared to prior proof-of-concept BCIs that often rely on more resolved 64-128 channel EEG or invasive neural recording modalities and utilize much more training data yet still yield variable results. With extended training data and adaptive incorporation of incoming neural data, future work could further enhance decoding accuracy.

Despite these encouraging results, several factors currently limit the direct applicability of our findings. First, the observed effect sizes may be less robust in real-world, multi-speaker environments that are more complex than those tested experimentally. Second, while our participant cohort spanned a range of hearing-loss profiles and attention abilities representative of the end users most likely to benefit from auditory enhancement, a larger sample reflecting this demographic would further strengthen the conclusions [39], [40]. This breadth increases confidence that the observed effects are not limited to a highly selected subgroup. Third, the system requires active engagement and sustained attention from the user, which can fluctuate with fatigue or cognitive load. Forth, the current implementation relies on a set of 24 wet-gel EEG electrodes to detect attentional signals, which may not yet be feasible for everyday use. Extending this approach to real-world applications will require optimizing electrode configurations, exploring dry and wireless EEG recording systems, lengthening training duration, and ensuring reliable decoding under variable listener effort and environmental conditions. To substantiate our interpretations, it would be necessary to include a separate control group that receives only sham or no feedback throughout their session to demonstrate the absence of session-wide effects. Within the discussion and supplemental materials, we have offered recommendations for future implementations of the training paradigm, which encompass guidelines for participant inclusion, suggestions for improved decoder training, and the design of future neurofeedback paradigms. In summary, our findings demonstrate that closed-loop neurofeedback can selectively suppress neural tracking of distracting speech. This proof-of-concept provides a foundation for developing attention-guided training paradigms and hearing aids for speech perception, though further work is required to scale the approach for everyday practical use.

## V. CONCLUSION

We employed real-time auditory attention decoding to facilitate a closed-loop neurofeedback training paradigm. This study was driven by the need to assist listeners who encounter difficulties in producing robust neural attentional measures suitable for utilization by an attention decoder. We devised a closed-loop neurofeedback system which provided feedback on a listener’s decoded attention strength, manifesting as a strengthened attenuation level for the unattended talker in the scene. We proposed three distinct hypotheses concerning improved attention throughout the session. Through the employment of AAD-derived neural tracking measures, we ascertained that participants exhibited decreased neural tracking of unattended talkers in the presence of neurofeedback. This work provides a comprehensive account of the system and protocol design process, elucidates the interpretations and limitations of the findings, and offers insights that may be integrated into future multi-session AAD-driven neurofeedback training paradigms.

## Supplementary Material

supp1-3623842

## Figures and Tables

**TABLE 1.** Neural tracking implications of attention decoder-derived metrics across the session, illustrating how changes in decoding metrics correspond to changes in listeners’ neural tracking of the attended and unattended talkers.

Change in Decoder Metric	Neural Tracking Implications
Increased ${\mathrm{corr}}_{Att,Att}$	Enhanced Attended Talker
Increased ${\mathrm{corr}}_{Att,Una}$	Ambiguous
Increased ${\mathrm{corrDiff}}_{\mathrm{Att}}$	Enhanced Attended Talker
Increased ${\mathrm{acc}}_{Att}$	Enhanced Attended Talker
Decreased ${\mathrm{corr}}_{\mathrm{Una},\mathrm{Una}}$	Suppressed Unattended Talker
Decreased ${\mathrm{corr}}_{Una,Att}$	Ambiguous
Decreased ${\mathrm{corrDiff}}_{\mathrm{Una}}$	Suppressed Unattended Talker
Decreased ${\mathrm{acc}}_{\mathrm{Una}}$	Suppressed Unattended Talker
