# Supplementary material for: A Brain-Computer Interface for Improving Auditory Attention in Multi-Talker Environments
Source: IEEE Access. Author manuscript; Available in PMC 2025 Nov 20. (PMC12629276; doi:10.1109/access.2025.3623842)
Supplement: supp1-3623842 [file NIHMS2122215-supplement-supp1-3623842.pdf]

# A Brain-Computer Interface for Improving Auditory Attention in Multi-Talker Environments

Stephanie Haro, Christine Beauchene, Thomas F. Quatieri, and Christopher J. Smalt

**Corresponding author:** Christopher J. Smalt
Christopher.Smalt@ll.mit.edu

# 1 Supplementary Materials

## 1.1 Audiometric Testing

Pure-tone audiometric testing was conducted using headphones in a sound-treated booth, employing the Wireless Automated Hearing Test System (WAHTS) developed by Creare LLC., Hanover, NH. The automated audiogram assessed pure-tone thresholds at frequencies of [0.125, 0.250, 0.5, 1, 2, 4, 8] kHz. The cohort of participants exhibited a range of hearing abilities. Audiometric thresholds were utilized to calculate the four-frequency pure-tone average across 500 Hz, 1 kHz, 2 kHz, and 4 kHz. Among the participants, seventeen exhibited a four-frequency pure-tone average equal to or above -10 decibel hearing-level (dB HL), while five participants had a mean pure-tone average of -36.1 dB HL. While typical studies might exclude individuals with a pure-tone average above -30 dB HL, our approach included these participants in the initial analysis to encompass the range of abilities that the neurofeedback system aims to address (Figure 1A).

The correlation between participants' audiometric ability and their capacity to accurately answer questions related to the attended story immediately following the preceding trial was significant (rho = 0.53, p = 0.02) (Figure 1B). This finding suggests that participant audiometric thresholds could influence the accuracy of speech perception in scenarios involving competing talkers, thereby affecting comprehension responses. Alternatively, it may imply that participants with more pronounced audiometric deficits may have more limited cognitive capacity, affecting their ability to track attention and recall details from the attended speaker's stream simultaneously. It has been established that individuals with hearing impairments exhibit higher difficulty ratings in competing speech tasks [1] and greater attention decoding accuracy compared to individuals with normal hearing [2]. To mitigate variability across measures, we recommend incorporating listener audiometric assessments and age into consideration, given the documented associations between auditory health and attention decoding measures [2]. We propose that individuals with a four-frequency pure tone average of 30dB HL (500Hz, 1kHz, 2kHz, 4kHz) and/or those using hearing aids should be examined separately from normal-hearing participants in future exploratory studies due to the potential variability they introduce to the results [2].

## 1.2 Pupillometry Measurements

A Tobii eye tracking bar recorded pupil diameter measurements at a sampling rate of 90 Hz. Before the commencement of the neurofeedback paradigm, a slow sinusoidally varying light calibration task was administered to document the pupil diameter response to changes in luminance [3]. The obtained pupil diameter measurements were participanted to artifact rejection, blink removal, and subsequently low-pass filtered at 10 Hz offline. We observed that participant's reactivity of their pupils to a light-based task was correlated with the attended decoder accuracy achieved during the session (rho = 0.53, p = 0.01), as shown in Figure 1C. This should be researched further to determine if this can be used as a hearing-independent participant characteristic that can signal attention difficulties. Furthermore, if a future study can recruit a cohort with a balanced age or audiometric composition, we advise that correlation-based analyses should be conducted to elucidate the relationships between participant characteristics (hearing abilities, age, and pupil light response), listener task-related pupil diameter, EEG power band measures, and attention neural tracking measures. We also recommended that future studies on training paradigms explore how improvements in attention decoding are associated with participants' cognitive capacity to undergo a training paradigm and the temporal plasticity of their decoder over the course of the session.

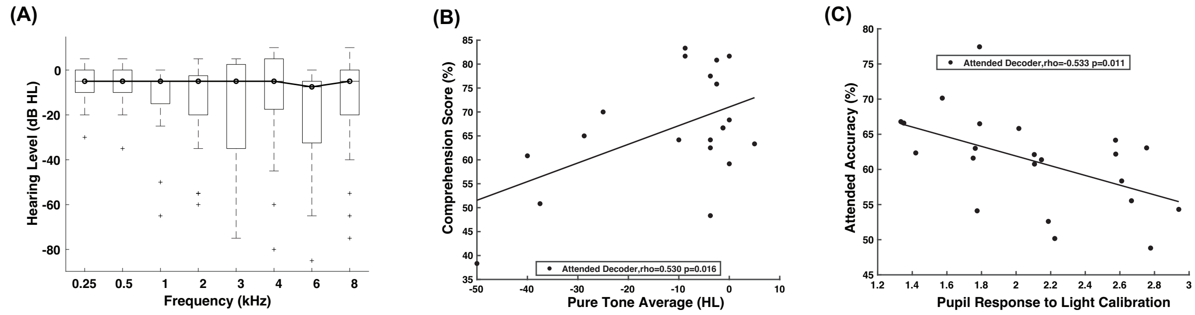


Figure 1: (A) Median pure-tone audiogram for the 22 included participants illustrates the spectrum of hearing ability that was captures in cohort. (B) Comprehension score plotted against pure-tone average. A participant's ability to correctly answer questions about the attended story was correlated to their audiometric ability. (C) A participant's decoded attention accuracy is correlated with how reactive their pupil was to a pre-session light-based calibration task

## 1.3 Causal Filter Design

Causality is defined as a property of signals that delineates a mathematical operation utilizing only past samples, devoid of any future points [4]. This contrasts with the application of the term causality in the field of neuroscience, where it pertains to a network of interconnected cortical processes wherein one process instigates another [5]. In this study, the concept of causality is employed in the context of signals and systems. AAD involves the use of two signal modalities: electroencephalography (EEG) and an audio stimulus representation. Certain groups engaged in the development of offline attention decoding algorithms participant their cortical data to extensive non-causal pre-processing to optimize EEG data quality entering the model, and to compute stimulus features that are non-viable in real-time systems. Although this approach is justifiable for the purpose of creating highly effective attention decoders, it results in the breach of causality across numerous pre-processing and model training phases. In prior AAD research, we also engaged in the use of non-causal, computationally demanding EEG pre-processing and audio envelope operations that do not fulfill the current causal requirements [6, 7]. Through employing a pilot cohort of participants involved in a standard open-loop attention task, we verified that the causal pre-processing pipeline implemented here indeed attained equivalent least-squares decoding accuracy as did the non-causal pre-processing pipeline utilized in our earlier publications [6, 7].

To enable the system to function in a real-time environment, it was imperative that the system's filters and other pre-processing components be executed causally, precluding any reliance on future samples relative to the current sample. In contrast, offline filtering is frequently achieved in a non-causal manner with filters applied in both forward and backward directions, thereby eliminating the group delay associated with the specific filter used. Offline filters are typically implemented as infinite-impulse-response (IIR) filters due to the reduced number of frequency taps required to produce a filter frequency response with specified sharpness and attenuation. Nonetheless, IIR filters present a significant challenge as they exhibit a frequency-dependent group delay [4], indicating that each frequency component of the unfiltered signal may experience varying group delays en route to becoming its filtered counterpart. This frequency-dependent group delay complicates the management of cumulative latencies arising from various pre-processing steps. Conversely, finite-impulse-response (FIR) filters yield a constant group delay frequency response, allowing the repositioning of the delayed filtered signal through a temporal shift not feasible with an IIR filter. Consequently, all filters employed in the pre-processing pipeline are FIR filters that possess a non-frequency-dependent latency. The FIR filter was implemented causally utilizing the Matlab filter function. Moreover, the system was required to 500 ms long data segments rather than prolonged, continuous data recordings. Consequently, when processing 500 ms data segment, the filters utilize the initial conditions preserved from the previous filter's implementation 500 ms earlier. Employing the previous filter implementation's values for subsequent filtering

operations mitigates edge effects each time the filter is applied. For attention decoding purposes, both EEG and audio signals were participanted to downsampling to 100Hz to expedite computation. Downsampling refers to generating a smoothed, lower-sampled version of the original signal, unlike decimation which entails sub-sampling the original signal to reduce the sampling rate. A downsampling function was devised that incorporates a causally-implemented FIR low pass filter as well.

## 1.4 Real-time Data Acquisition and Stimulus Level Augmentation

The closed-loop system must rapidly access data during the experiment to perform operations with minimal delay. Signal acquisition software utilizing lab-streaming-layer (LSL) technology represents a significant advancement, facilitating access to data streams as they are recorded in real-time [8, 9]. In our application, there is a requirement for real-time, time-synchronized access to the recorded EEG and audio signals to derive an attention decision signal. This is achieved by establishing LSL-based communication inlet channels with the EEG audio playback stream within a Matlab instance dedicated to data acquisition and analysis. Data is streamed into Matlab in 500 ms intervals, undergoing causal pre-processing and decoding. Subsequently, real-time decoded attention is transmitted via a communication outlet channel to a stimulus presentation instance of Matlab at a frequency of one decoded decision every 500 ms. The stimulus level of the unattended talker is incrementally augmented at a rate of 2 Hz based on the listener performance.

## 1.5 EEG Pre-Processing

EEG pre-processing was minimized to facilitate the evaluation of decoding results against real-time decoding implementations that constrain pre-processing to enhance speed [10]. Consequently, no blink or artifact removal was performed on the EEG data due to the time and data demands of these processes. As an example, independent component analysis (ICA), a traditional method for removing blinks from EEG, was deemed unsuitable as it requires the entire session's data from the participant to identify components related to blinks. The EEG data was bandpass filtered between [2,8] Hz to align with the delta and theta bands that correspond to the slower rhythmic structure of word onsets and syllable rates in speech [11]. This bandpass filtering was executed at the original sampling rate of 500 Hz, using an FIR filter with a passband frequency range from [2,8] Hz, a stopband frequency range of [1,16] Hz, and a stopband attenuation of 60dB. Due to the real-time data acquisition and casual pre-processing requirements, both the EEG and audio envelope were required at a sampling rate of 100 Hz to make the data manageable in size. Accordingly, the EEG data was causally downsampled to 100 Hz. The accumulated pre-processing latency is 1.02 s i.e. 102 samples at 100 Hz (Figure 2). For training purposes, the EEG data was z-scored using all 10 minutes of 24-channel data to compute the mean and standard deviation values for z-scoring. When a new data segment is processed by the decoder, it is z-scored using the mean and standard deviation derived from training to preserve causality.

## 1.6 Audio Pre-Processing

For the audio envelope computation, a simple yet effective method was utilized, as the preferred iterative method is non-causal and significantly computationally intensive for real-time applications [12]. In alignment with the MTRF toolbox methods [13] that have been used decode with much success, the audio envelope was defined as the square root of the squared audio waveform (Eq. 1 and Eq. 2). A multi-step downsampling process was then applied to bring down the audio envelope to 100 Hz. The downsampled envelope was bandpass filtered with parameters matching the EEG bandpass filter but designed for a 100 Hz sampling rate. The net latency contribution from the audio pre-processing pipeline is is 1.073 s at 100 Hz i.e. 107 samples at 100 Hz (Figure 2). For every 500 ms segment, we addressed modality pre-processing latency mismatches by realigning the downsampled signal onsets and trimming samples to ensure identical durations. There may be a data loss of 10-50 ms (1-5 samples at 100 Hz) at the end of each 500 ms segment processed through the pipeline. Compared to the 5 s delay associated with the 10 s correlation window used, added up with the approximately 1.1 s delay associated with causal pre-processing, this few sample loss is deemed negligible for the closed-loop system implementation [7, 14].

$$\mathbf{Env}_{Att} = \sqrt{(\mathbf{Aud}_{Att})^2} \tag{1}$$

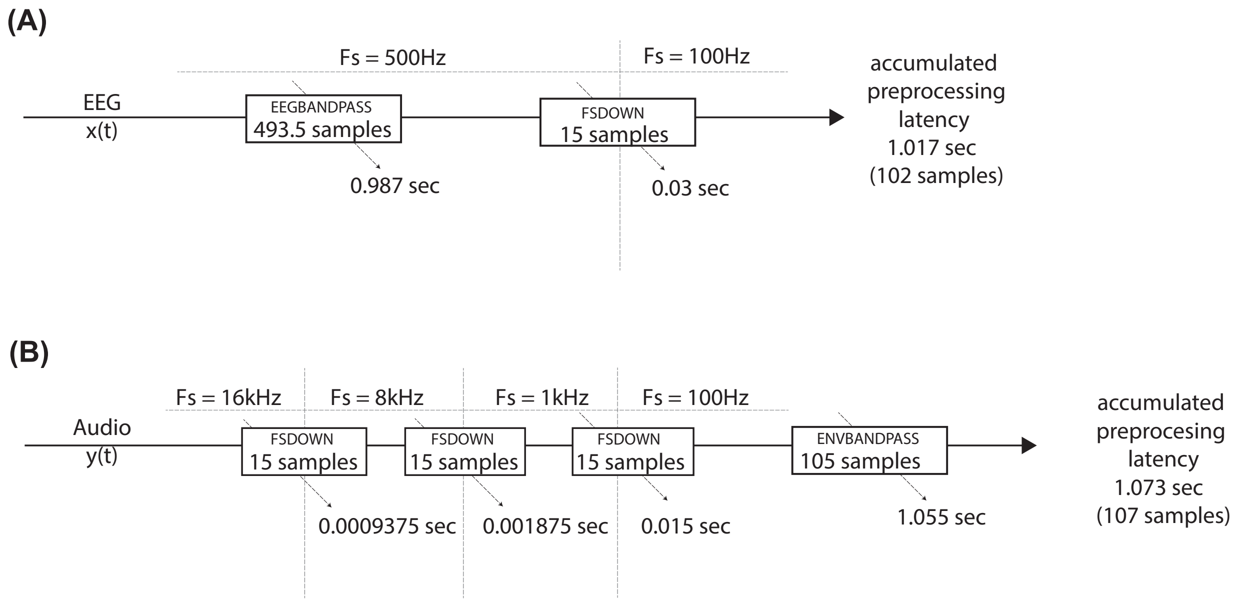


Figure 2: Causal pre-processing introduced latencies when the data was passed through these filters. These latencies are particularly important when modality streams undergo different pre-processing steps and the data needs to time aligned before it can be used in a computational model of attention.

$$\mathbf{Env}_{Una} = \sqrt{(\mathbf{Aud}_{Una})^2} \tag{2}$$

## 1.7 Unattended Talker Decoder Derivations

In Eq. 3, the unattended talker decoder, $W_{Una}$, was solved using L2 regularized least squares using the same training set of data as the attended decoder, $N_{Tr}$, but the talker envelope being solved is different. In a manner analogous to the attended decoder, employing the unattended decoder, $W_{Una}$, on the window of neural data, $N_{Test}$, yields the predicted unattended talker envelope, $\widehat{env}_{Una}$ (Eqs. 4).

$$\mathbf{W}_{Una} = (\mathbf{N}_{Tr}{}^T\mathbf{N}_{Tr} + \lambda I)^{-1}(\mathbf{N}_{Tr}{}^T\mathbf{Env}_{Una,Tr}) \tag{3}$$

$$\widehat{\mathbf{env}}_{Una} = \mathbf{N}_{Test}\mathbf{W}_{Una} \tag{4}$$

The process of decoding the unattended talker parallels the method used for the attended talker decoder but employs the unattended decoder weights, $W_{una}$. Metric $corr_{Una,Una}$ evaluates the neural tracking of the decoded unattended envelope to the unattended talker envelope, $env_{Una}$. Meanwhile, $corr_{Una,Att}$ assesses the neural tracking performance of the decoded unattended envelope to the attended talker envelope. Metric $corrDiff_{Una}$ measures the dissimilarity between $corr_{Una,Una}$ and $corr_{Una,Att}$, highlighting the strength of the decoder's neural tracking of the unattended talker that is not shared with the attended talker envelope. Additionally, $acc_{Una}$ quantifies the proportion of samples during which the decoded unattended envelope, $\widehat{env}_{Una}$, exhibits stronger tracking of the unattended talker envelope in comparison to the attended talker envelope.

$$\mathbf{corr}_{Una,Una} = corr(\widehat{\mathbf{env}}_{Una}, \mathbf{env}_{Una}) \tag{5}$$

$$\mathbf{corr}_{Una,Att} = corr(\widehat{\mathbf{env}}_{Una}, \mathbf{env}_{Att}) \tag{6}$$

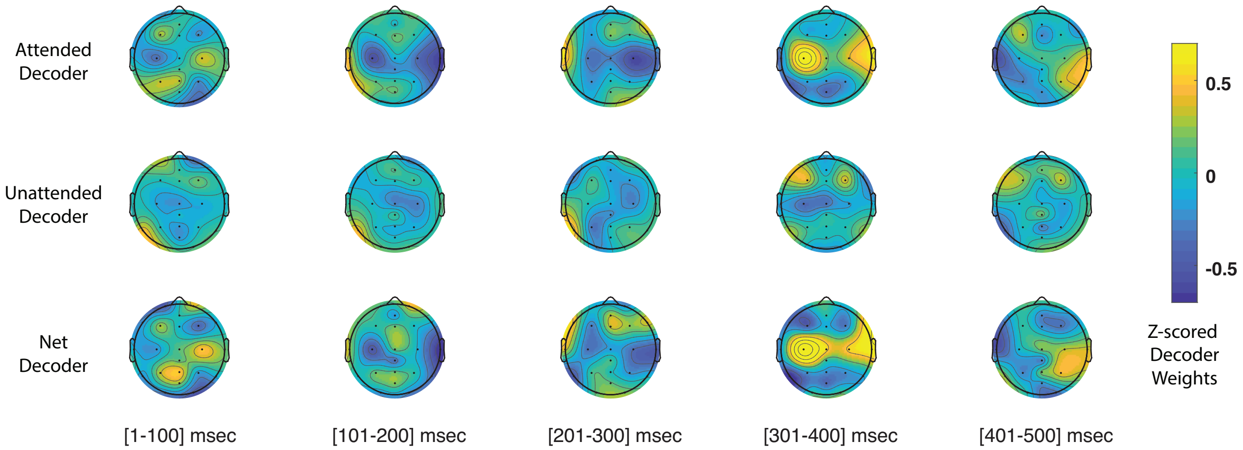


Figure 3: Grand mean z-scored decoder topography weights show channel and latency dependent differences between the attended and unattended talker decoder weights. (A) Attended decoder topography (B) Unattended decoder topography (C) The net talker decoder topography highlights positive attended decoder differences across the 100 ms segment proceeding 0 ms, 300 ms, and 400 ms.

$$\mathbf{corrDiff}_{Una} = \mathbf{corr}_{Una,Una} - \mathbf{corr}_{Una,Att} \tag{7}$$

$$acc_{Una} = mean(\mathbf{corrDiff}_{Una} > 0) \tag{8}$$

## 1.8 Auditory Attention Decoder Topographies

The net decoder weights were computed to discern the variations between attended and unattended decoder weights (Eq. 11). Each participant's decoder weights were z-scored before calculating the grand average, thereby normalizing the decoders within individual participants (Eq. 9 - 12). The grand-mean z-scored attended and unattended decoder weights are depicted in Figure 3. Decoder weight trajectories across the EEG channels at various time points can be visualized using an EEG channel topography. Given that the decoder possesses an excessively fine temporal resolution for visualization across weights and topographies over time, local time averages of 100 ms were computed to downsample the decoder weights into five discrete time points for representation on the EEG topographies. The attended and unattended talker decoder weights exhibit differences at several time lags within the 500 ms EEG window. Specifically, at 100 ms, 400 ms, and 500 ms, the attended talker decoder displays more positive weights than the unattended talker decoder. These variations are emphasized in the bottom row of weight topographies. It is imperative not to attempt to interpret the spatial distribution of the decoders, as the weights do not consistently reflect the stimulus encoding locations and temporal characteristics [11].

Grand mean attended decoder across participants, $W_{Att,Grand}$, is computed as follows where $\mu_{Att,n}$ and $\sigma_{Att,n}$ are the mean and standard deviation of matrix, $W_{Att,n}$ :

$$\mathbf{W}_{Att,Grand} = \sum_{i=1}^{N} \frac{(\mathbf{W}_{Att,n} - \mu_{Att,n})}{\sigma_{Att,n}} \tag{9}$$

Grand mean attended decoder across participants, $W_{Una,Grand}$, is computed as follows where $\mu_{Att,n}$ and $\sigma_{Una,n}$ are the mean and standard deviation of matrix, $W_{Una,n}$ :

$$\mathbf{W}_{Una,Grand} = \sum_{i=1}^{N} \frac{(\mathbf{W}_{Una,n} - \mu_{Una,n})}{\sigma_{Una,n}} \tag{10}$$

Grand mean attended decoder across participants, $W_{Net,Grand}$, is computed as follows where $\mu_{Att,n}$ and $\sigma_{Net,n}$ are the mean and standard deviation of matrix, $W_{Net,n}$ :

$$\mathbf{W}_{Net} = \mathbf{W}_{Att} - \mathbf{W}_{Una} \tag{11}$$

$$\mathbf{W}_{Net,Grand} = \sum_{i=1}^{N} \frac{(\mathbf{W}_{Net,n} - \mu_{Net,n})}{\sigma_{Net,n}} \tag{12}$$

# References


[1] Søren A Fuglsang, Jonatan Märcher-Rørsted, Torsten Dau, and Jens Hjortkjær. Effects of sensorineural hearing loss on cortical synchronization to competing speech during selective attention. *Journal of Neuroscience*, 40(12):2562–2572, 2020.

[2] Lien Decruy, Jonas Vanthornhout, and Tom Francart. Hearing impairment is associated with enhanced neural tracking of the speech envelope. *Hearing Research*, page 107961, 2020.

[3] Tepring Piquado, Derek Isaacowitz, and Arthur Wingfield. Pupillometry as a measure of cognitive effort in younger and older adults. *Psychophysiology*, 47(3):560–569, 2010.

[4] Alain de Cheveigné and Israel Nelken. Filters: when, why, and how (not) to use them. *Neuron*, 102(2):280–293, 2019.

[5] Anil K Seth, Adam B Barrett, and Lionel Barnett. Granger causality analysis in neuroscience and neuroimaging. *Journal of Neuroscience*, 35(8):3293–3297, 2015.

[6] Gregory Ciccarelli, Michael Nolan, Joseph Perricone, Paul T Calamia, Stephanie Haro, James O'Sullivan, Nima Mesgarani, Thomas F Quatieri, and Christopher J Smalt. Comparison of two-talker attention decoding from eeg with nonlinear neural networks and linear methods. *Scientific reports*, 9(1):1–10, 2019.

[7] Stephanie Haro, Hrishikesh M Rao, Thomas F Quatieria, and Christopher J Smalt. Eeg alpha and pupil diameter reflect endogenous auditory attention switching and listening effort. *European Journal of Neuroscience*, 2022.

[8] C Kothe, D Medine, C Boulay, M Grivich, and T Stenner. Lab streaming layer (lsl)-a software framework for synchronizing a large array of data collection and stimulation devices. *The Swartz Center for Computational Neuroscience Software. Available online: https://code. google. com/archive/p/labstreaminglayer/(accessed on 29 February 2020)*, 2012.

[9] Christopher Smalt, Hrishikesh Rao, and Gregory Ciccarelli. mit-ll/signal-acquisition-modules-for-lab-streaming-layer: v1.0. Mar 2021.

[10] Emina Alickovic, Thomas Lunner, Fredrik Gustafsson, and Lennart Ljung. A tutorial on auditory attention identification methods. *Frontiers in neuroscience*, 13:153, 2019.

[11] Marlies Gillis, Jana Van Canneyt, Tom Francart, and Jonas Vanthornhout. Neural tracking as a diagnostic tool to assess the auditory pathway. *Hearing Research*, page 108607, 2022.

[12] Rachelle L Horwitz-Martin, Thomas F Quatieri, Elizabeth Godoy, and James R Williamson. popelka2016a vocal modulation model with application to predicting depression severity. In *2016 IEEE 13th International Conference on Wearable and Implantable Body Sensor Networks (BSN)*, pages 247–253. IEEE, 2016.

[13] Michael J Crosse, Giovanni M Di Liberto, Adam Bednar, and Edmund C Lalor. The multivariate temporal response function (mtrf) toolbox: a matlab toolbox for relating neural signals to continuous stimuli. *Frontiers in human neuroscience*, 10:604, 2016.

[14] Simon Geirnaert, Tom Francart, and Alexander Bertrand. An interpretable performance metric for auditory attention decoding algorithms in a context of neuro-steered gain control. *IEEE Transactions on Neural Systems and Rehabilitation Engineering*, 28(1):307–317, 2019.
